# Global investment targets for malaria control and elimination between 2016 and 2030

**DOI:** 10.1136/bmjgh-2016-000176

**Published:** 2017-05-16

**Authors:** Edith Patouillard, Jamie Griffin, Samir Bhatt, Azra Ghani, Richard Cibulskis

**Affiliations:** 1 Global Malaria Programme, World Health Organization, Geneva, Switzerland; 2 Swiss Tropical and Public Health Institute, Basel, Switzerland; 3 Universität Basel, Basel, Switzerland; 4 School of Mathematical Sciences, Queen Mary University of London, Mile End Road, London, UK; 5 Medical Research Council Centre for Outbreak Analysis and Modelling, Department of Infectious Disease Epidemiology, Imperial College London, London, UK

## Abstract

**Background:**

Access to malaria control interventions falls short of universal health coverage. The Global Technical Strategy for malaria targets at least 90% reduction in case incidence and mortality rates, and elimination in 35 countries by 2030. The potential to reach these targets will be determined in part by investments in malaria. This study estimates the financing required for malaria control and elimination over the 2016–2030 period.

**Methods:**

A mathematical transmission model was used to explore the impact of increasing intervention coverage on burden and costs. The cost analysis took a public provider perspective covering all 97 malaria endemic countries and territories in 2015. All control interventions currently recommended by the WHO were considered. Cost data were sourced from procurement databases, the peer-reviewed literature, national malaria strategic plans, the WHO-CHOICE project and key informant interviews.

**Results:**

Annual investments of $6.4 billion (95% uncertainty interval (UI $4.5–$9.0 billion)) by 2020, $7.7 billion (95% UI $5.4–$10.9 billion) by 2025 and $8.7 billion (95% UI $6.0–$12.3 billion) by 2030 will be required to reach the targets set in the Global Technical Strategy. These are equivalent to annual investment per person at risk of malaria of US$3.90 by 2020, US$4.30 by 2025 and US$4.40 by 2030, compared with US$2.30 if interventions were sustained at current coverage levels. The 20 countries with the highest burden in 2015 will require 88% of the total investment.

**Conclusions:**

Given the challenges in increasing domestic and international funding, the efficient use of currently available resources should be a priority.

**Key questions**

**What is already known about this topic?**

- Extending malaria control measures to universal health coverage targets is a key challenge. Insufficient investment contributes to the current intervention coverage gaps and malaria burden.
- New global targets for malaria control and elimination have been adopted for the 2016–2030 period. A methodology to estimate the cost of accelerating progress towards reaching these targets is required in order to monitor funding progress and identify shortfalls over the next 15 years.
- Previous estimates of global resource needs for malaria control and elimination have not considered malaria transmission dynamics and the effectiveness of combinations of interventions.

**What are the new findings?**

- Under the Global Technical Strategy for malaria 2016–2030, annual investment targets per person at risk of malaria are estimated at US$3.90 by 2020, US$4.30 by 2025 and US$4.40 by 2030.
- The total investment need for malaria control and elimination is not expected to decrease before 2030, reflecting in part population growth in currently high burden countries and the costs of surveillance in countries near elimination.
- Increasing investments in malaria control and elimination efforts remains highly cost-effective at around US$12 per additional malaria case averted. Savings in malaria treatment costs, which are likely to be financed in large part by domestic sources, are expected from 2025 onwards, with the greatest savings in the current highest burden countries.

**Recommendations for policy**

- Given the challenges in rapidly increasing domestic and international funding for malaria and health more generally, ensuring the efficient use of currently available resources to maximise value for money should be a priority.

## Introduction

Substantial gains have been made in the fight against malaria since the beginning of the millennium. Between 2000 and 2015, global case incidence has fallen by an estimated 41% and mortality rates by 62%.^1^ In 2015, the number of countries reporting <1000 malaria cases was three times the number doing so in 2000.^2^ Most of these gains have been directly attributed to increasing coverage of core malaria control measures, notably in sub-Saharan Africa where transmission is the most intense.^3^ Despite this, the implementation of malaria control activities falls short of universal health coverage (UHC) targets and malaria continues to pose a major public health challenge in countries where it remains endemic. In 2015, around 3 billion people were estimated to be at risk of acquiring malaria, with 212 million cases and 429 000 related deaths estimated to occur. Malaria also poses a serious economic burden on health systems and economic development, particularly in the most affected countries, which are also often the poorest.^1^ ^4^

The Global Technical Strategy (GTS) for malaria 2016–2030 was developed to accelerate progress towards malaria elimination. The GTS targets a global reduction of at least 90% in malaria case incidence and mortality rates, and elimination in at least 35 countries by 2030. Intermediate milestones include global reductions in disease burden of at least 40% by 2020 and 75% by 2025, and elimination in at least 10 and 20 countries by 2020 and 2025, respectively. The GTS highlights the need to increase access to malaria interventions including vector control, chemoprevention in high-risk populations, and prompt access to diagnosis and treatment of malaria in health facilities or in the community. Surveillance is also recognised as a core intervention to target resources where they are most needed and to detect and eliminate the remaining cases and foci of malaria.^5^ Mathematical modelling suggests that currently the WHO recommended interventions would need to reach 90% coverage by 2030 to approach the target of a 90% reduction in disease burden and malaria elimination.^6^

A critical factor determining the potential to reach these targets will be the financial resources invested in malaria from domestic and international sources over the next 15 years. Since 2003, global investments in malaria control and elimination have increased nearly 20-fold, reaching US$2.9 billion in 2015.^1^ This, however, represents only half of the US$5.6 billion estimated to be required annually to reach the coverage targets of malaria control interventions in the 2008 Global Malaria Action Plan.^7^ With the endorsement of the GTS by the World Health Assembly in 2015, this paper generates new global investment targets for malaria 2016–2030. Investment targets are estimated using a malaria transmission model that enables intervention coverage levels to be varied, and the impact on programme costs and malaria burden to be explored.

## Methods

### Scope and perspective

We estimated the total and incremental levels of investments required annually to increase coverage of malaria control interventions to 90% by 2030 in order to reach the GTS goals and targets. The analysis was undertaken from the perspective of the public healthcare provider covering the 97 malaria endemic countries and territories in 2015. All malaria control interventions recommended by the WHO in 2015 were considered, including long-lasting insecticidal nets (LLINs) and complementary vector control interventions (eg, indoor residual spraying, larval source management), seasonal malaria chemoprevention in children (SMC), intermittent preventive treatment of pregnant women (IPTp), diagnostics by blood testing and treatment of confirmed cases. Surveillance activities included routine epidemiological and entomological information systems in all countries, supplemented at low levels of malaria transmission by a locally tailored response to every detected malaria infection or the occurrence of outbreaks. Our analysis does not consider the deployment of new interventions.

### Scenarios

We examined two scenarios that differed in the levels of intervention coverage. The counterfactual scenario was defined as sustaining the current coverage of control measures over the 2016–2030 period. The second scenario, ‘accelerate’, envisages coverage of the same interventions reaching 90% by 2030 (table 1).

**Table 1:** Description of scenarios

Scenario	Intervention	Baseline	2020 coverage targets	2025 coverage targets	2030 coverage targets
1. Sustain	All	Maintain coverage of all interventions at 2011–2013 levels			
2. Accelerate	Vector control LLIN IRS	Means of 2011–2013 country-specific coverage	80% with LLIN replaced every 3 years	90% with LLIN replaced every 2 years	Maintain coverage
	Other measures	10% additional coverage with complementary control measures for resistance management			
	SMC	0%	80%	95%	Maintain
	IPTp	0%	80%	90%	Maintain
	Blood tests, using RDTs or microscopy	20%			90%
		10% G6PD testing			90%
	Treatment of uncomplicated cases	2013 country-specific coverage	90% at public facilities, 50% in communities	.	75% community based treatment
	Treatment of severe cases	100% hospitalised cases treated with quinine	100% hospitalised cases treated with injectable artesunate	50% severe cases with rectal artesunate in communities	75% severe cases with rectal artesunate in communities

The 2011–2013 levels were assumed until 2015 and were then scaled up; vector control modelled as LLIN.

LLIN, long-lasting insecticidal treated nets; IRS, indoor residual spraying; LSM, larval source management; SMC, seasonal malaria chemoprevention; IPTp, intermittent preventive treatment of malaria in pregnant women; RDT, rapid diagnostic test; G6PD, glucose-6-phosphate dehydrogenase deficiency.

Under ‘accelerate’, all interventions were scaled up to the same level of coverage everywhere regardless of the predicted changes in local transmission between 2016 and 2030. Annual total investment targets were estimated as the sum of all intervention costs. Incremental investment targets were calculated as the difference between the costs of increasing coverage to 90% and the costs of sustaining interventions at their current coverage levels.

We also explored the sensitivity of our estimates by stratifying intervention coverage targets by projected malaria transmission levels over the whole study period. For this, we developed four subscenarios or stratification approaches. The first was to stop increasing intervention coverage in countries that can achieve a 90% reduction in malaria incidence before reaching the coverage targets. The second assumed that preventive interventions would be scaled back locally 3 years after local elimination was attained. The third assumed that coverage of non-malarial fever (NMF) testing would be reduced from target levels to 10% of the population at risk (PAR) once malaria transmission fell below 1 case per 5000 people per year. The fourth was to combine these three stratification approaches.

### Intervention coverage and impact estimates

#### Vector control

For 80 countries with stable *Plasmodium falciparum* transmission in 2010,^8^ estimates of the PAR to be covered by vector control were obtained by overlaying population estimates from the Global Rural–Urban Mapping Project^9^ with estimates of parasite prevalence in children aged 2–10 at a 1 km^2^ resolution.^8^ These estimates were then aggregated to country level and scaled to match the United Nations (UN) world population estimates for the same year.^10^ For 17 other countries with low levels of *P. falciparum* transmission (most of these were reported as eliminating malaria in 2014^2^), PAR estimates were obtained from the World Malaria Report 2014.^11^ The UN world population projections stratified by urban and rural status were used to extrapolate the PAR forward by year to 2030 (see online supplementary figure S1).

10.1136/bmjgh-2016-000176.supp1 supplementary data

Estimates of LLIN coverage for countries outside Africa in 2013 were obtained from the World Malaria Report 2014. For countries in Africa, we used estimates of LLIN coverage from a model combining delivery data from manufacturers with data on household coverage from Demographic Health Surveys (DHS) and other population-based surveys.^12^ Projections forward for LLIN coverage were made as outlined in table 1. For LLINs, these targets are based on the proportion of households protected by LLINs. To translate this into the required number of nets to be distributed, we used outputs from the model by Bhatt *et al*^12^ under the ‘minimised overallocation’ model. For the strategy of insecticide resistance management, we assumed that 10% of the population would be covered with complementary control measures.

#### Chemoprevention

Seasonal malaria chemoprevention (SMC) was costed by calculating the PAR under 5 years of age in the areas of the Sahel region of Africa in which it is recommended.^13^ UN projections of the age distribution of the population over time were used, stratified by urban–rural settings. We assumed zero coverage in 2013 reflecting the recent recommendation of this intervention. For chemoprevention during pregnancy (IPTp), we estimated the number of eligible women (those in their second or third trimester of pregnancy) using projections of total live births (estimated using PAR of malaria and UN crude birth rates) plus spontaneous pregnancy loss (ie, miscarriages and stillbirths) after the first trimester^14^ in the high transmission settings in which it is currently recommended. We assumed zero per cent coverage of the four IPTp doses in 2013.^1^ Coverage targets from 2016 to 2030 were as outlined in table 1.

#### Diagnosis and treatment

Projections of the likely impact of the ‘accelerate’ scenario on *P. falciparum* case incidence for the 80 countries with stable transmission were derived from a mathematical model of *P. falciparum* transmission.^6^ For the 17 other countries that were not included in the modelling since levels of *P. falciparum* transmission were too low, we assumed that case incidence was maintained at 2015 levels for costing purposes. This conservative approach allows for financial resources that may be required to maintain surveillance once elimination is achieved.

Since *P. vivax* models are not sufficiently well developed to provide comparative projections, the modelled numbers of uncomplicated *P. falciparum* cases among populations under and over 5 years were used to estimate the number of uncomplicated *P. vivax* cases based on the reported ratio of *P. falciparum* to *P. vivax* cases in each country.^2^ The rate of severe disease for clinical *P. vivax* was assumed to be two-thirds that of *P. falciparum* and the death rate 40%.^6^ For both *P. falciparum* and *P. vivax*, we additionally included treatment costs associated with false-positive tests. This was estimated using the reported specificity rate for current rapid diagnostic tests (RDTs).^15^ All modelled case estimates were multiplied by the proportion of patients with malaria receiving appropriate care at public facilities or in the community.^2^ Treatment dosage for populations aged under and over 5 years was assumed to follow the WHO recommended treatment guidelines.^16^

The number of diagnostic tests for malaria cases was estimated using the projected uncomplicated and severe malaria case incidence, assuming diagnosis is undertaken either using microscopy or an RDT based on diagnostic use for suspected cases reported by countries.^11^ Coverage rate projections were made as outlined in table 1. For the number of diagnostics for NMFs, we calculated the average rate of NMFs in under-5 children by subtracting the model-based estimates of malaria cases from estimates of under-5 fevers derived from demographic health surveys and management information systems, giving an estimate of 3.50 NMF episodes per child per year. We assumed one fever episode per year for older age populations. Finally, we assumed that 20% of NMFs received a malaria test in 2015 and that this scaled up linearly to 90% by 2030. Glucose-6-phosphate dehydrogenase deficiency testing was included for *P. vivax* cases assuming 10% of cases would be tested in 2016 increasing linearly to 90% by 2030.

#### Surveillance

For all countries, surveillance was assumed to include routine epidemiological and entomological components and regular malaria indicator surveys. As countries move to lower levels of transmission and hence towards elimination, additional surveillance costs were included for case and foci investigations and classification and/or proactive case detection with activity implementation stratified by locally specific annual malaria parasite incidence^6^ (table 2).

**Table 2:** Surveillance assumptions over the malaria control-elimination spectrum

Surveillance activities	Coverage rate	Stratification criteria
Epidemiological and entomological surveillance	100% of population at risk	All areas
Operational research	1 household and 1 health facility malaria survey modules every 3–5 years	All areas
Case/foci investigation and classification activities	15% of cases	4≤API<5
	30% of cases	3≤API<4
	50% of cases	2≤API<3
	70% of cases	1≤API<2
	90% of cases	0.5≤API<1
	100% of cases	0.5<API
Proactive case detection	10% of population at risk	0.5≤API<1
	5% of population at risk	0.5<API

API, annual parasite incidence.

### Estimates of intervention unit costs

Commodity procurement prices were sourced from international databases or from expert consultations for commodities with no prices available. Cost data on freight and insurance, in-country delivery and surveillance were obtained from the published literature, available national malaria strategic plans and from National Malaria Control Programmes (NMCP) reports for the World Malaria Reports. For all countries, patient delivery cost estimates for treatment at health facilities were sourced from the WHO CHOICE.^17^ table 3 and the online supplementary file provide more details on the data sources.

**Table 3:** Median cost parameters and assumed distribution for probability sensitivity analysis, by intervention (constant 2014 US$)

Interventions	Median (IQR) base case	Distribution for probabilistic sensitivity analysis	Sources
*LLIN*			
Procurement price, per net	$3.72 ($1.50)	γ	18
Freight and insurance mark-up	20% (20%)	γ	19
In-country delivery mark-up	48% (20%)	γ	20
*Complementary vector control* (costing modelled on IRS)			
Estimated average total cost per person protected per year	$4.24 ($2.25)	γ	21
*IPTp using SP*			
Procurement price per dose of 3 SP tablets	$0.17 ($0.20)	γ	18
In-country delivery mark-up	15% (20%)	γ	19 22
Patient delivery cost per dose of 3 tablets	$0.44 ($0.20)	γ	22
Number of doses	4	Point estimate	Key informant interview
*SMC*			
Procurement cost per SP+amodiaquine course	$1.44 ($1.50)	γ	23
In-country delivery mark-up through public facility, outreach clinics and village health workers	70% (20%)	γ	24–27
Number of rounds	3	Point estimate	Key informant interview
*Diagnosis*			
Procurement cost per rapid diagnostic test/microscopy slide	$0.60 ($0.30) income groups 1, 2 $0.80 ($0.50) income groups 3, 4	γ	28 29
Number of fevers per person per year	3.50 in U5, 1 in 5+	Point estimate	Authors' estimation
Procurement cost per G6PD deficiency test	$5.00 ($7.00)	γ	Key informant interview
In-country delivery mark-up (public health facility)	15% (20%)	γ	
In-country delivery and patient delivery mark-up (communities)	40% (20%)	γ	30–33
*Treatment* Procurement cost per dose of ACT	$0.48 ($0.20) U5 in WB income groups 1, 2	γ	28
	$0.63 ($0.20) 5+ in WB income groups 3, 4		
	$1.30 ($0.40) in U5 in WB income groups 3, 4		
	$1.70 ($1.00) in 5+ in WB income groups 3, 4		
Procurement cost per dose of chloroquine and primaquine	$0.83 ($0.20) for U5 and $3.33 ($1.50) in 5+ in WHO WPR and SEAR regions	γ	18
	$0.53 ($0.20) for U5 and $2.13 ($1.50) in 5+ elsewhere		
Procurement cost per dose of ACT and primaquine	$1.07 ($0.50) for U5 and $3.52 ($1.50) in 5+ in WHO WPR and SEAR regions	γ	
	$1.36 ($1.50) for U5 and $4.69 ($1.50) in 5+ elsewhere		
Procurement cost per dose of quinine	$0.91 ($0.20) in U5	γ	
	$3.63 ($1.50) in 5+		
Procurement cost per dose of injectable artesunate	$5.36 ($1.50) in U5		
	$14.4 ($1.50) in 5+		
In-country delivery mark-up (public health facility)	15% (20%)	γ	19
In-country delivery and patient delivery of diagnosis and treatment (as part of community management)	55% (20%)	γ	34–37
Outpatient visit and inpatient stay (range across levels of care/facility type)	Country-specific cost estimates	Uniform	17
Estimated number of hospital bed day stay for severe malaria episode	3	Point estimate	Key informant interviews
*Surveillance*			
Cost of epidemiological and entomological surveillance per person at risk, and malaria surveys	$0.05 ($0.03)	γ	34–39
		
Case investigation, per case	$290 ($100)	γ	
Proactive case detection, per case	$3.12 ($2.00)	γ	
Wastage mark-up	10% (10%)	γ	19 22

ACT, artemisinin combination therapy; LLIN, long-lasting insecticidal treated nets; G6PD, glucose-6-phosphate dehydrogenase deficiency; IPTp, intermittent preventive treatment in children; IRS, indoor residual spraying; SMC, seasonal malaria chemoprevention in children; SP, sulfadoxine-pyremithamine; WB, World Bank; WPR, Western Pacific Region; SEAR, South-East Asia Region; U5, under-5.

### Estimating investment targets

The average unit cost of implementing an intervention can vary with the scale at which the intervention is delivered and with the range of other interventions implemented alongside it. (Dis)economies of scale or scope describe a situation where average unit costs (increase) decrease with increasing programme reach or scope. At very high levels of intervention coverage, average unit costs rise with the increasing cost of reaching the last few populations. Managing the process of intensifying malaria control interventions when scaling up several interventions at the same time could also produce diseconomies of scope if current infrastructures are overwhelmed, or economies of scope by piggybacking on underused capacity.^40^ Malaria control interventions are likely to be expanded in settings with different levels of infrastructure, a situation that would affect the incremental costs of increasing intervention coverage. Scaling-up processes are also likely to use different pathways that would most likely impact costs and cost structures.^41^ However, reliable evidence on how cost and cost structure vary with coverage levels and across different settings in low-income and middle-income countries is extremely limited, notably for malaria control interventions.^20^ In this context, we chose not to impose cost functions in this global analysis^42^ and conducted extensive uncertainty analysis to capture potential variations in unit costs during our investment target estimation.

### Handling uncertainty

We conducted probabilistic uncertainty analysis using Monte Carlo simulations to determine a 95% uncertainty range for the investment need estimates. Cost parameters were assigned a γ probability distribution informed by median cost estimates and IQRs from the literature, except for outpatient and inpatient CHOICE cost range estimates, for which a uniform distribution was used (table 3). Cost parameters were varied simultaneously to obtain 1000 costs simultaneously combined with 50 stochastic realisations of case incidence projections from the mathematical model of *P. falciparum* transmission.^6^ The mean unit costs and uncertainty intervals (UI) across the 1000 cost estimations for core malaria control interventions are reported in the online supplementary file.

## Results

**Figure 1:**
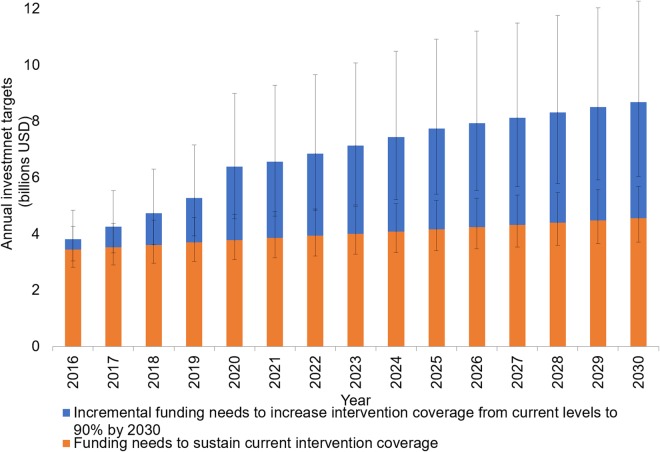
Global annual investment targets for malaria control and elimination under sustain and accelerate scenarios with 95% uncertainty intervals (constant 2014 US$).

Reducing malaria case incidence and mortality risk by 90% globally by 2030 is estimated to require annual investments of $6.4 billion (95% UI $4.5–$9.0 billion) by 2020, $7.7 billion (95% UI $5.4–$10.9 billion) by 2025 and $8.7 billion (95% UI $6.0–$12.3 billion) by 2030 (figure 1). This translates to an investment need of US$101.8 billion (95% UI $72.6–$142.0 billion) over 15 years, equivalent to a 40% increase in investments compared with sustaining coverage (US$60.1 billion, 95% UI $49.2–$74.7 billion). In the initial 5 years, the required costs increase most rapidly as levels of coverage are scaled up from current levels to 80%. In addition, the substantial projected population growth in the affected countries further increases the need. From 2020 onwards, the lower linear increase is due to increasing coverage from 80% to 90%.

We estimated that 88% of the total investment will be required by the 20 countries with the highest burden in 2015. The majority of the total cost was estimated in Africa (63.1%) where malaria transmission is most intense, with the remaining in the Asia and Pacific regions (30.3%), Europe and the Middle East (5.3%) and Central and South America (1.4%).

Overall, the annual investment target per person at risk amounts to US$3.90 by 2020, US$4.30 by 2025 and US$4.40 by 2030, compared with, on average, US$2.30 if interventions were to be sustained at their current coverage levels over the 15 years. Reaching these investment targets are expected to avert an additional 171 million cases and 646 100 deaths by 2020.^6^

**Figure 2:**
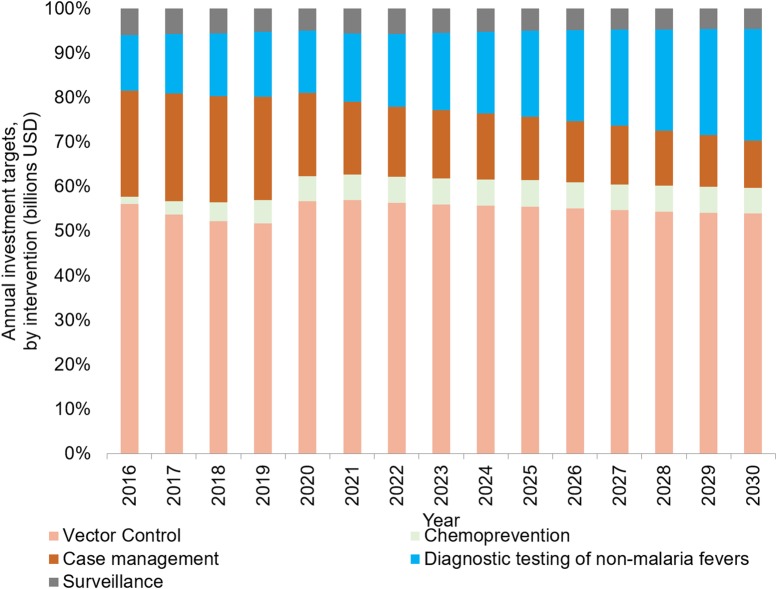
Per cent of global annual investment targets for malaria control and elimination under the accelerate scenario, by intervention (constant 2014 US$).

**Figure 3:**
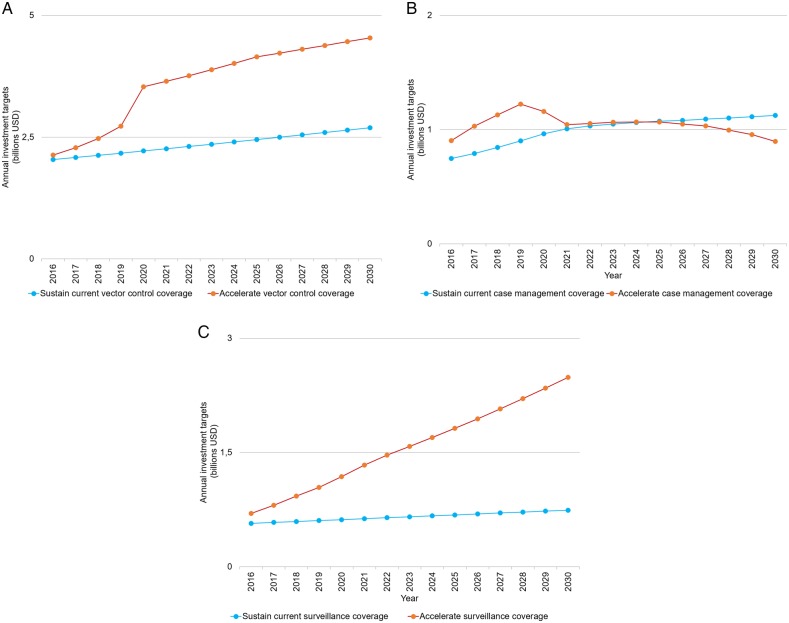
(A) Per cent of global annual investment targets for vector control interventions under sustain and accelerate scenarios (constant 2014 US$). (B) Global annual investment targets for diagnostics and treatment of malaria cases in the public sector (health facility and community levels) under sustain and accelerate scenarios (constant 2014 US$). (C) Global annual investment targets for surveillance activities (including diagnostics of non-malaria fevers) under sustain and accelerate scenarios (constant 2014 US$).

We predict that the majority of investments will be required for prevention activities, notably vector control (55%, 95% UI 44%–64%) and chemoprevention (5.6%, 95% UI 1.4%–10.7%). Substantial investments will also be required for diagnosis of NMFs (20%, 95% UI 9%–32%), malaria case management (15%, 95% UI 10%–23%) and surveillance (5%, 95% UI 3%–6%; figure 2). These proportions are, however, expected to change over the 15-year period as the impact of increasing intervention coverage reduces the malaria burden (figure 3A–C). Specifically, the costs of malaria treatment are predicted to decline from 2020 onwards, representing a decrease in the absolute malaria burden and a proportionate decrease that outweighs the underlying population growth (figure 3B). Savings in case management costs are expected from 2025 onwards, with the greatest savings in the current highest burden countries. In contrast, surveillance costs are projected to increase as more countries progress towards elimination (figure 3C). In addition, we project an increase in the costs associated with testing NMFs as an increasing proportion of tests confirm a negative malaria diagnosis (figure 3C).

**Figure 4:**
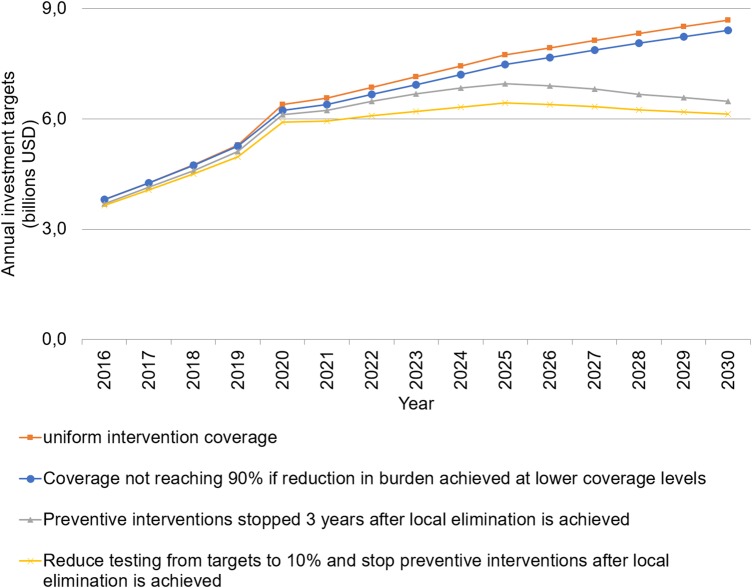
Sensitivity of estimated global annual investment targets to four stratification approaches under the accelerate scenario (constant 2014 US$).

We assessed the sensitivity of our base-case estimates by considering alternative scenarios for the resource need as malaria elimination is approached. Investment targets were, as expected, most sensitive to variations in the assumed need for vector control once local elimination has been achieved, with an estimated reduction of 23% in annual investments by 2030 (US$6.4 billion, 95% UI 4.5–9.0 billion, or US$3.30, 95% UI $2.28–$4.56, per person at risk) if vector control is removed 3 years following local elimination. Reducing universal malaria testing of NMFs to a target of 10% of NMFs after local elimination is projected to further reduce needs to US$6.1 billion (95% UI 4.3–8.6 billion), or US$3.10 ($2.20–$4.36) per person at risk by 2030. Other approaches marginally decreased funding requirements (figure 4).

## Discussion

Investments in malaria control and elimination activities were estimated at around $2.9 billion in 2015.^1^ To accelerate transmission reductions and consequently further reduce malaria-associated morbidity and mortality, we estimate that global annual investments in malaria will need to increase to $6.4 billion (95% UI $4.5–$9.0 billion) by 2020, $7.7 billion (95% UI $5.4–$10.9 billion) by 2025 and $8.7 billion (95% UI $6.0–$12.3 billion) by 2030. This translates to an annual cost of intensifying malaria control globally ranging between US$3.90 and US$4.40 per person at risk depending on the level of intervention coverage achieved. This falls within the broad range of per capita estimates published previously.^43^

Despite the large sums required to achieve the goals set out in the GTS, the intervention mix remains highly cost-effective. Under the strategy costed here, Griffin *et al*^6^ estimate, on average, that an additional 220 million cases will be averted annually. With this estimated to cost nearly $102 billion over the 15 years compared with $60 billion to sustain interventions at their current level, this translates to a cost per additional case averted of around $12. Furthermore, as many countries move towards elimination and subsequently progressively discontinue prevention interventions, the global costs are predicted to plateau towards the end of this 15-year period. Thus, accelerating progress towards elimination will see a move towards reducing the financial burden of malaria globally.

The costing approach developed here provides a methodology to monitor progress over the next 15 years and to identify shortfalls in the funding required to achieve the goals set out in the GTS. Our approach to estimate a global price tag for the global malaria strategy uses dynamic modelling to underpin forward projections of impact. A key benefit of this approach is that our investment targets account for malaria transmission dynamics and the effectiveness of combinations of interventions rather than of interventions implemented in silos.

We assume a slower but more realistic rate of increase in intervention coverage compared with previous studies, which projected reaching UHC within 2 years of intense scale-up.^7^ ^44^ We predict increasing investment needs throughout the 15-year period compared with contemporary studies that assume decreasing needs from 2025 as large geographical areas achieve elimination.^45^ ^46^ This difference is in part due to the substantial population growth projected over the next 15 years in high burden countries, such that, in our modelling, the reduced cost associated with elimination in a number of countries is counterbalanced at the global level by the increasing population size in those countries remaining endemic. The only scenario in which we estimate a reduction in global needs is if universal vector control coverage is scaled back once local elimination occurs. However, scaling back vector control in areas where local transmission has been interrupted is not recommended without a thorough evaluation of the epidemiological characteristics and capacities of health systems to detect and respond to potential reintroduction and resurgence,^47^ which in themselves may introduce other costs.

Our approach has a number of limitations. First, we did not include any constraints in the capacity of countries to scale up to high levels of coverage across all interventions. In reality, in many resource-poor settings, the lack of human resources or/and their poor productivity^48^ are likely important determinants of the effect and costs of malaria control strategies. This may therefore limit the potential impact of interventions. Furthermore, we only included the direct costs to the health system, and thus while we captured additional personnel time, associated human capital and infrastructure spending needed to increase the capacity of health systems was not included. Equally, we did not capture the savings to health systems that could be obtained from reducing the burden of malaria and hence potentially freeing capacity to treat other conditions. Second, we did not include the costs of near-term innovations that would most likely be required before 2030 to reach the GTS goals because of the uncertainty around the nature of these innovations and their implementation costs. Third, we did not estimate the additional costs of research and development for malaria as these have been estimated elsewhere (an additional US$673 million per year (range US$524–US$822 million)).^5^ Finally, we focused on the Sustainable Development Goal period and did not attempt to provide estimates post-2030 because of the increasing uncertainty in the type, effectiveness and costs of interventions.

Despite the substantial increase in financing towards malaria control and elimination over the past 15 years, we expect challenges in attaining the investment targets for 2016–2030. Relatively optimistic assumptions suggest that international and domestic contributions may increase to $3.8 billion by 2020,^11^ which implies a funding gap of $2.6 billion in this year. Only through significant increases in domestic and international funding, and in particular through economic growth and greater commitment to internationally agreed targets, could the current gap in financing begin to be bridged.^11^ While economic growth generates additional resources which can contribute to increasing government expenditure for health,^49^ ^50^ countries at the highest risk of malaria are often the most resource-constrained and international funding sources are likely to continue playing a significant role in funding malaria interventions in the 2016–2030 period. Interlinkages between progress towards malaria elimination and economic wealth also imply that as countries get wealthier, they face graduation from donors' funding while successful malaria elimination requires predictable sustained funding to reach and sustain malaria-free status. These factors indicate that global malaria control and elimination face major challenges in the Sustainable Development Goal financing landscape. Ensuring the efficient use of currently available resources to maximise value for money should therefore be a priority, with a focus on the most effective interventions targeted to the populations most in need.
