# Supplementary material for: Global investment targets for malaria control and elimination between 2016 and 2030
Source: BMJ Glob Health. 2017 May 16;2(2):e000176. doi: 10.1136/bmjgh-2016-000176 (PMC5584487; doi:10.1136/bmjgh-2016-000176)
Supplement: supplementary data [file bmjgh-2016-000176supp001.pdf]

Supplementary information to Manuscript "Global investment targets for malaria control and elimination 2016-2030"

1. **Projected population at risk of malaria**

Figure S1: Projected population residing in areas at risk of *P.falciparum* malaria transmission 2016-2030

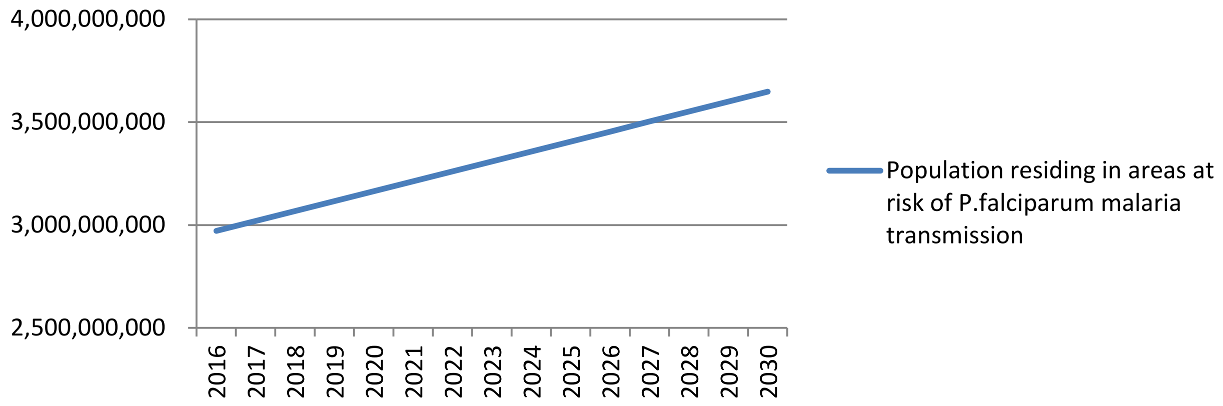

2. **Commodity procurement cost data**

Median and inter-quartile range of procurement costs were calculated using transaction prices sourced from international databases. The cost of an LLIN was estimated by calculating the median and interquartile range of 54 price observations of rectangular LLINs provided by the WHO Global Service Centre procurement database in July 2014. RDT and ACT procurement prices were sourced from the Global Fund's Price and Quality Reporting database, by reviewing all purchase order transactions recorded to have taken place between 01 September 2010 and 01 July 2014. We assumed freight and insurance costs were not included in these transactions as recommended by the Global Fund. For SMC amodiaquine and sulphadoxine-pyrimethamine procurement prices were sourced from the UNICEF procurement database by calculating the average cost of under-5 year and under-1 year treatment regimens. Procurement price data for other anti-malarial drugs were sourced from the WHO procurement database. Drug procurement costs were inflated for freight and insurance, which were assumed to add a median cost of 20% (IQR 20).

3. **In-country distribution cost data**

In-country distribution costs refer to the costs of distributing commodities to the point of service delivery. Wastage was assumed to represent 10% of all commodities procured, except for LLIN for which inefficiencies were accounted for in the volume of LLIN to be distributed to reach a given

coverage level. For interventions delivered at routine facilities, distribution to the point of service delivery was assumed to add 15% (IQR 20).

Vector control. In-country delivery of LLIN was assumed to add an additional median of 48% (IQR 20) to the procurement price.[1] The median add-on cost included personnel (21%), overheads (6%), capital (2%), distribution/transport (15%), storage and other costs (7%). For complementary vector control measures, we assumed an average cost per person protected per year of $4.24 based on the estimated costs of IRS available from the President Malaria Initiative (PMI) over the 2008-2012 period. [2] We used IRS cost estimates in our calculations rather than estimates of other vector control interventions because of the limited evidence available on the implementation cost of the latter (e.g. larval source management). However, we recognize that larval source management may be used as a complementary measure to LLIN for the management of insecticide resistance.

SMC. In-country delivery using routine clinics, outreach or trekking services and/or village workers was estimated to add on average 70% (IQR 20) on top of procurement prices for personnel, supervision, training and management costs.[3-6]

Diagnostic and treatment. When delivered in the community as part of ICCM, in-country delivery was estimated to add 40% (IQR 20) on top of procurement prices, including personnel, training, supervision and management costs[7-9].

4. Patient delivery cost data

At facility level, the costs of treating clinical and severe malaria cases were estimated using cost data on outpatient visit or inpatient hospital stay sourced from the WHO CHOICE database[10]. The cost of an outpatient visit was estimated using country specific CHOICE data on the cost of an outpatient visit at rural (lower bound cost estimate) or urban (higher bound cost estimate) primary care facilities. Similarly, the cost of an admission was estimated using country specific CHOICE data assuming a 3-day bed-stay at primary (lower bound cost estimate) or tertiary (higher bound cost estimate) hospitals. Deaths were also costed assuming a 1.5-day hospital bed-stay. When for a given country CHOICE estimates were not available, the missing value was imputed by the median cost for countries with available data from the same World Bank income group. When dispensed at community level as part of ICCM, the patient delivery cost was assumed to add 55% (IQR) on top of procurement cost inflated for in-country distribution. [7-9 11]

5. Estimated mean unit costs and uncertainty intervals

Table 4 Mean unit costs and uncertainty intervals (UI) for core malaria control interventions (constant USD 2014)

Intervention	Mean unit cost (95% UI)
**LLIN**	
- per net delivered	$6.87 ($3.36-$11.90)
- per person protected per year	$1.27 ($0.62-$2.20)
**SMC**	
- per round/course	$1.24 ($0.14-$3.68)
- per child protected per year	$3.72 ($0.42-$11.04)
**IPTp**	
- per pregnant woman protected	$2.68 ($1.27-$4.76)
**Diagnostic***	
- per rapid diagnostic test	$1.06 ($0.41-$2.02)
- per G6PD test	$6.95 ($0.31-$23.34)
**Treatment***	
- per uncomplicated *P falciparum* case treated	$0.83 ($0.38-$1.50) in under-fives<br>$2.28 ($1.09-$4.73) in older populations
- per uncomplicated *P vivax* case treated	$1.28 ($0.70-$2.20) in under-fives<br>$4.60 ($2.32-$7.99) in older populations
- per severe malaria case treated	$8.96 ($5.07-$15.16) in under-fives<br>$23.82 ($16.27-$36.71) in older populations

Note - LLIN: long lasting insecticidal treated nets; SMC: chemoprevention in children; IPTp: intermittent preventive treatment in children; G6PD: Glucose-6-phosphate dehydrogenase deficiency; *excludes patient delivery cost

References

1. White MT, Conteh L, Cibulskis R, et al. Costs and cost-effectiveness of malaria control interventions--a systematic review. Malar J 2011;10:337.
2. Abbott M, Johns B. PMI IRS Country Programs: comparative cost analysis. Bethesda, MD: Abt Associates Inc., 2013.
3. Conteh L, Patouillard E, Kweku M, et al. Cost effectiveness of seasonal intermittent preventive treatment using amodiaquine & artesunate or sulphadoxine-pyrimethamine in Ghanaian children. PLoS One 2010;5(8):e12223.
4. Patouillard E, Conteh L, Webster J, et al. Coverage, adherence and costs of intermittent preventive treatment of malaria in children employing different delivery strategies in Jasikan, Ghana. PLoS One 2011;6(11):e24871.
5. Temperley M, Mueller DH, Njagi JK, et al. Costs and cost-effectiveness of delivering intermittent preventive treatment through schools in western Kenya. Malar J 2008;7:196.
6. Bojang KA, Akor F, Conteh L, et al. Two strategies for the delivery of IPTc in an area of seasonal malaria transmission in the Gambia: a randomised controlled trial. PLoS Med 2011;8(2):e1000409.
7. Jarrah Z, Lee A, Wright K, et al. Costing of integrated community case management in Rwanda. http://www.tractionproject.org/sites/default/files/MSH_Costing_of_Integrated_Community_Case_Management_Analysis_Report_Rwanda.pdf, 2013.
8. Jarrah Z, Lee A, Wright K, et al. Costing of integrated community case management in Malawi. http://www.msh.org/sites/msh.org/files/msh_costing_of_integrated_community_case_management_analysis_report_malawi.pdf, 2013.
9. Jarrah Z, Wright K, Suraratdecha C, et al. Costing of integrated community case management in Senegal. http://www.msh.org/sites/msh.org/files/msh_costing_of_integrated_community_case_management_analysis_report_sene.pdf, 2013.
10. World Health Organization. CHOICE: Choosing Interventions that are cost-effective, 2008.
11. Collins D, Jarrah Z, Gilmartin C, et al. The costs of integrated community case management (iCCM) programs: a multi-country analysis. J Glob Health 2014;4(2).
